# Influence of the Non-Schmid Effects on the Ductility Limit of Polycrystalline Sheet Metals

**DOI:** 10.3390/ma11081386

**Published:** 2018-08-08

**Authors:** Mohamed Ben Bettaieb, Farid Abed-Meraim

**Affiliations:** 1 Université de Lorraine, CNRS, Arts et Métiers ParisTech, LEM3, F-57000 Metz, France; Mohamed.BenBettaieb@ensam.eu; 2 Laboratory of Excellence on Design of Alloy Metals for Low-mAss Structures (DAMAS)—Université de Lorraine, F-57073 Metz, France

**Keywords:** crystal plasticity, non-Schmid effects, Taylor multiscale scheme, localized necking, bifurcation theory

## Abstract

The yield criterion in rate-independent single crystal plasticity is most often defined by the classical Schmid law. However, various experimental studies have shown that the plastic flow of several single crystals (especially with Body Centered Cubic crystallographic structure) often exhibits some non-Schmid effects. The main objective of the current contribution is to study the impact of these non-Schmid effects on the ductility limit of polycrystalline sheet metals. To this end, the Taylor multiscale scheme is used to determine the mechanical behavior of a volume element that is assumed to be representative of the sheet metal. The mechanical behavior of the single crystals is described by a finite strain rate-independent constitutive theory, where some non-Schmid effects are accounted for in the modeling of the plastic flow. The bifurcation theory is coupled with the Taylor multiscale scheme to predict the onset of localized necking in the polycrystalline aggregate. The impact of the considered non-Schmid effects on both the single crystal behavior and the polycrystal behavior is carefully analyzed. It is shown, in particular, that non-Schmid effects tend to precipitate the occurrence of localized necking in polycrystalline aggregates and they slightly influence the orientation of the localization band.

## 1. Introduction

Despite the significant progress accomplished in the modeling of the mechanical behavior of metallic materials, the study of localized necking in thin metal sheets remains an active research topic for both academic and industrial communities. This research area has been initiated in the pioneering contributions of Keeler and Backofen [1], and Goodwin [2], who have introduced the representation by a forming limit diagram (FLD) as a characterization of the initiation of localized necking in thin metal sheets. For each strain path ranging from uniaxial tension to equibiaxial tension, the in-plane principal strains, which are associated with the incipience of localized necking, are reported on the forming limit diagram. Considering the practical complexity related to the experimental determination of FLDs (precise identification of the moment of the onset of strain localization, scatter in the experimental data…), as well as their high cost (a lot of experimental tests required to build a complete FLD…), important efforts have been devoted to the development of several alternative theoretical and/or numerical prediction models. These models are generally based on the coupling between a localization criterion used to predict the onset of localized necking and a constitutive model describing the evolution of the mechanical fields. Among the most known localization criteria used in the literature, one can quote the initial imperfection approach, which was developed initially by Marciniak and Kuczynski [3], the bifurcation theory initiated by Rice in [4,5], and the energy criterion of instability of a deformation process formulated by Petryk et al. [6,7,8]. Despite its large popularity, the initial imperfection approach has a main drawback: the over-sensitivity of its predictions (in terms of limit strain) to the amount of initial imperfection (which may be viewed as a nonphysical parameter). By contrast, the use of the bifurcation theory does not need any additional parameter (such as the initial imperfection factor). Furthermore, the formulation of the bifurcation theory is based on sound mathematical considerations, and its numerical implementation is relatively easy. For these reasons, the bifurcation theory is used in the current paper to detect the incipience of strain localization. For elastic-plastic constitutive frameworks with an associated plastic flow rule and a smooth yield surface, it has been demonstrated (see, e.g., Reference [5]) that the bifurcation approach is unable to predict material instability at a realistic strain level in the range of positive strain paths. Consequently, the prediction of localized necking at realistic strain levels requires the use of constitutive models exhibiting some destabilizing effects. When phenomenological constitutive models are coupled with the bifurcation theory, destabilizing phenomena may be introduced either by including damage-induced softening effects [9,10] or by deviating the plastic flow rule from normality [11,12]. Despite their popularity, the phenomenological models are not able to accurately capture the effects of some essential physical and microstructural mechanisms (initial and induced crystallographic and morphologic textures, crystallographic structure, dislocation density evolution…) on some important in-use properties (strength, formability…). These limitations have motivated researchers to use multiscale models in the prediction of FLDs. In the current contribution, a multiscale model has been coupled with the bifurcation theory to predict the incipience of localized necking in polycrystalline aggregates. In this multiscale model, the Taylor scheme is used to determine the macroscopic behavior of the polycrystalline aggregates from that of their constituents (single crystals). The mechanical behavior at the single crystal scale follows a finite strain rate-independent formulation. Compared to the rate-dependent formulation that has been widely used to predict strain localization [13,14], the rate-independent one is more appropriate for the simulation of cold forming processes, where viscous effects are limited. In the majority of the previous rate-independent contributions [15,16,17,18], the plastic flow is modeled by the classical Schmid law [19]. In this case, the destabilizing mechanism required to predict bifurcation localization at realistic limit strain levels is an obvious consequence of the crystal plasticity multi-slip and the associated yield surface vertex effects, which is taken into account by using this classical Schmid law. The effect of a regularization of this Schmid law (by substituting the vertices at the yield surface by rounded corners) on the prediction of the ductility limit has been recently analyzed in [20]. It has been demonstrated that the limit strains predicted in the range of positive strain paths are unrealistically high when the regularized version of the Schmid law is used to model the plastic flow at the single crystal scale. Although the classical Schmid law is widely accepted, various experimental observations made on BCC (Body Centered Cubic) metallic materials, such as molybdenum [21], tungsten [22], or tantalum [23], have revealed that this classical law is not always able to accurately describe the plastic flow. Indeed, most BCC single crystals have 24 slip systems, but the slip planes are not ideally close-packed. The 1/2〈111〉 screw dislocations in BCC metals have a non-planar core structure that spreads on three $\left\{1\bar{1}0\right\}$ planes. This causes non-Schmid effects (effects that are not considered by the classical law), in which stresses developing on planes and directions other than those on the primary slip systems will influence dislocation motion [24]. Note that the non-Schmid effects have been introduced in the modeling of the mechanical behavior of steel materials in at least three recent studies [24,25,26]. In these contributions, two particular steel grades have been studied: the three-phase QP980 and dual-phase DP980 steels. The reason for the choice of these steels is principally related to the fact that the deformation of the ferrite phase in both grades shows non-negligible non-Schmid behavior. These non-Schmid effects are incorporated in a generalized version of the Schmid law [27]. In such a generalization, the single crystal yield function includes stress components other than the Schmid stress, which results in non-associated plastic flow (i.e., non-normality). The main motivation behind the current investigation is to carefully analyze the implications of deviations from the classical Schmid law (by the incorporation of the non-Schmid effects) on the constitutive modeling of both BCC single crystals and polycrystalline aggregates. Then, the impact of these deviations on the onset of localized necking in thin metal sheets and on the orientation of the associated localization band is investigated. It will be especially demonstrated that the non-Schmid effects tend to precipitate the occurrence of strain localization in polycrystalline aggregates.

The paper is organized as follows. Section 2 gives the theoretical framework on which the prediction of localized necking in BCC polycrystalline aggregates is based. Particular attention is paid in this section to the introduction of non-Schmid effects in the plastic flow. Section 3 outlines the numerical aspects related to the implementation of the equations that govern the prediction of the ductility limit of polycrystalline aggregates. In Section 4, various numerical results are provided, which illustrate the influence of the non-Schmid effects on the mechanical behavior and on the onset of localized necking. The conclusions drawn from this study are provided in Section 5. Finally, Appendix A provides the list of crystallographic slip systems for BCC single crystals.

Standard notations and conventions are used throughout:

▪ First, second, or fourth-order tensors are represented by bold-face letters and symbols (the order of which is indicated by the context).
▪ Scalar parameters and variables are designated by thin letters and symbols.
▪ Macroscopic (resp. microscopic) fields are designated by capital (resp. small) letters and symbols.
  $\dot{•}$ time derivative of •.
  ${•}^{\nabla }$ co-rotational derivative of •.
  ${•}^{T}$ transpose of •.
  •.• inner product.
  •:• double contraction product ($={•}_{ij}{•}_{ij}$ for the product between two second-order tensors, and
  ${•}_{ijkl}{•}_{kl}$ for the product between a fourth-order tensor and a second-order tensor).
  •×• vector product.
  det(•) determinant of tensor •.
  sgn(•) sign of •.

## 2. Theoretical Framework

In the following theoretical developments, an updated Lagrangian formulation will be used to express the different equations (constitutive equations and localization analysis). Let us consider a polycrystalline aggregate made of $N_{g}$ single crystals, which are initially randomly oriented. This aggregate is assumed to be representative of the studied thin metal sheet. To predict the ductility limit of the latter, the aggregate is submitted to uniform biaxial straining, where the in-plane components of the macroscopic velocity gradient G are known and defined as follows:

(1) $$G_{11}=1\;;\;G_{22}=\rho \;;\;G_{12}=G_{21}=0,$$

where *ρ* denotes the strain-path ratio, and it ranges between −1/2 (uniaxial tensile state) and 1 (equibiaxial tensile state). The out-of-plane components of G are unknown and should be deduced from the plane-stress condition in the direction normal to the plane of the sheet:

(2) $${\dot{N}}_{13}={\dot{N}}_{23}={\dot{N}}_{31}={\dot{N}}_{32}={\dot{N}}_{33}=0,$$

where $\dot{N}$ is the macroscopic nominal stress rate, which is related to the macroscopic velocity gradient G through the macroscopic tangent modulus L:

(3) $$\dot{N}=L:G.$$

The bifurcation criterion is used to numerically determine the onset of localized necking in the polycrystalline aggregate for the whole range of strain-path ratios (comprised between −1/2 to 1). This criterion asserts that strain localization occurs when the acoustic tensor becomes singular [5]. Hence, this criterion is defined by the following expression:

(4) $$\det(\vec{𝓝}.L^{\mathrm{PS}}.\vec{𝓝})=0,$$

where $\vec{𝓝}$ is the unit vector (lying in the plane of the sheet) normal to the localization band. Here, $\vec{𝓝}$ is taken equal to (cosθ,sinθ), where the orientation θ of the localization band is comprised between 0° and 90°. As to tensor $L^{PS}$, it represents the 2D macroscopic tangent modulus relating the in-plane components of the nominal stress rate tensor to the in-plane components of the velocity gradient. The analytical expression for the 2D tangent modulus $L^{PS}$ is derived from the general expression of the 3D tangent modulus by the classical relation [20]:

(5) $$\forall \;i,j,k,l=1,2:\;L_{ijkl}^{PS}=L_{ijkl}-\frac{L_{ij33}L_{33kl}}{L_{3333}}$$

Then, to check the occurrence of strain localization, the 3D macroscopic tangent modulus L should be determined by integrating the constitutive equations associated with the polycrystalline aggregate. In the current contribution, the Taylor multiscale scheme was used to determine the macroscopic mechanical behavior from the behavior of the microscopic constituents (the single crystals). This scheme was based on the assumption of the homogeneity of the strain field over the aggregate. Consequently, the microscopic velocity gradient g is equal to its macroscopic counterpart G. The macroscopic nominal stress rate $\dot{N}$ is obtained from its microscopic counterpart $\dot{n}$ by the averaging relation:

(6) $$\dot{N}=\frac{1}{V}\int_{V}\dot{n}(x)\;dx,$$

where V denotes the volume of the polycrystalline aggregate and x is a material point within this aggregate.

By combining the assumption of homogeneity of the strain field and Equation (6), one can deduce that the macroscopic tangent modulus L is related to its microscopic counterpart l (which relates $\dot{n}$ to g) by a relationship that is similar to Equation (6):

(7) $$L=\frac{1}{V}\int_{V}l(x)\;dx$$

Therefore, to compute the macroscopic tangent modulus L via Equation (7), the microscopic tangent modulus l for all individual grains should be first determined. To this end, the subsequent developments are dedicated to the derivation of the analytical expression of the microscopic tangent modulus.

The microscopic velocity gradient g is additively split into its symmetric and skew-symmetric parts, denoted d and w, respectively:

(8) g=d+w

The strain rate tensor d and the spin tensor w are decomposed into their elastic and plastic parts:

(9) $$d=d^{e}+d^{p}\;;\;w=w^{e}+w^{p}$$

The evolution of the rotation r of the lattice frame of the single crystal is expressed as a function of the elastic spin tensor $w^{e}$:

(10) $$\dot{r}.r^{T}=w^{e}\;$$

The elastic part of the behavior law is defined by the relation between the co-rotational derivative $k^{\nabla }$ of the Kirchhoff stress tensor and the elastic strain rate $d^{e}$:

(11) $$k^{\nabla }=C^{e}:d^{e}$$

where $C^{e}$ is the fourth-order elasticity tensor. Here, isotropic and linear elasticity is assumed. We recall that the Kirchhoff stress tensor k is defined by the following relation:

(12) k=j σ

where j is the Jacobian of the deformation gradient and σ is the Cauchy stress tensor.

The combination of Equations (11) and (12) gives:

(13) $$\sigma^{\nabla }+\sigma \;Tr(d)=C^{e}:d^{e}$$

The use of the co-rotational derivative $k^{\nabla }$ instead of the simple time derivative $\dot{k}$ of the Kirchhoff stress aims to satisfy the objectivity principle. These two derivatives are related by the following relation:

(14) $$\dot{k}=k^{\nabla }-k.w^{e}+w^{e}.k$$

The plastic deformation of the single crystal is assumed to be only due to the slip on the crystallographic planes. Thus, the plastic strain rate $d^{p}$ and the plastic spin $w^{p}$ can be expressed as follows:

(15) $$d^{p}=\sum_{\alpha =1}^{N_{s}}{\dot{\gamma }}^{\alpha }\;\mathrm{sgn}(\tau^{\alpha })\;R^{\alpha }\;;\;w^{p}=\sum_{\alpha =1}^{N_{s}}{\dot{\gamma }}^{\alpha }\;\mathrm{sgn}(\tau^{\alpha })\;S^{\alpha }$$

where:

▪ $N_{s}$ is the total number of slip systems.
▪ ${\dot{\gamma }}^{\alpha }$ is the absolute value of the slip rate of the αth slip system.
▪ $R^{\alpha }$ and $S^{\alpha }$ are respectively the symmetric and skew-symmetric part of the Schmid orientation tensor, which is defined as the tensor product ${\vec{m}}^{\alpha }⊗{\vec{n}}^{\alpha }$. Vectors ${\vec{m}}^{\alpha }$ and ${\vec{n}}^{\alpha }$, corresponding to BCC single crystals that we have used in the current work are listed in Appendix A.
▪ $\tau^{\alpha }$ is the resolved shear stress of the αth slip system, which is equal to $R^{\alpha }:\sigma$.

The plastic flow of the single crystal is modeled by a generalized version of the Schmid law [21]:

(16) $$\forall \alpha =1,\ldots ,N_{s}:\;\left\{ \begin{array}{l} \left|\tau^{\alpha }+\sum_{i=1}^{N_{ns}}a_{i}^{\alpha }\tau_{i}^{\alpha }\right|<\tau_{c}^{\alpha }\Rightarrow {\dot{\gamma }}^{\alpha }=0\\ \left|\tau^{\alpha }+\sum_{i=1}^{N_{ns}}a_{i}^{\alpha }\tau_{i}^{\alpha }\right|=\tau_{c}^{\alpha }\Rightarrow {\dot{\gamma }}^{\alpha }\geq 0 \end{array}\right..$$

where:

■ $\tau_{c}^{\alpha }$ is the critical shear stress of the αth slip system.
■ $\sum_{i=1}^{N_{ns}}a_{i}^{\alpha }\tau_{i}^{\alpha }$ is an additional term (compared to the classical Schmid law) used to capture the non-Schmid effects. Here, $\tau_{i}^{\alpha }$ and $N_{ns}$ denote the non-Schmid shear stresses and their number, respectively. As to $a_{i}^{\alpha }$, they represent material parameters, which can be determined by experimental tests or atomistic simulations [28]. For simplicity, we assume in the current contribution that $a_{i}^{\alpha }$ are the same for all of the slip systems, and we choose the following expansion for the term $\sum_{i=1}^{N_{ns}}a_{i}^{\alpha }\tau_{i}^{\alpha }$ [28]:
  (17) $$\sum_{i=1}^{N_{ns}}a_{i}^{\alpha }\tau_{i}^{\alpha }=\;a_{1}\sigma :({\vec{m}}^{\alpha }⊗{\vec{n}}_{1}^{\alpha })+a_{2}\sigma :[({\vec{n}}^{\alpha }\times {\vec{m}}^{\alpha })⊗{\vec{n}}^{\alpha }]+a_{3}\sigma :[({\vec{n}}_{1}^{\alpha }\times {\vec{m}}^{\alpha })⊗{\vec{n}}_{1}^{\alpha }]$$
  where vectors ${\vec{n}}_{1}^{\alpha }$ are enumerated in Appendix A (for the case of BCC single crystals).

Compared to other approaches given in the literature (see, for instance, References [28,29,30]) devoted to the study of the impact of the non-Schmid effects on the plastic flow of single crystals, Equation (17) seems to be the simplest expansion that we can consider.

By using the expression of the resolved shear stress $\tau^{\alpha }$ ($=R^{\alpha }:\sigma$) and Equation (17), the generalized Schmid law can be rewritten as follows:

(18) $$\forall \alpha =1,\ldots ,N_{s}:\;\left\{ \begin{array}{l} \left|\tau^{*\;\alpha }\right|<\tau_{c}^{\alpha }\Rightarrow {\dot{\gamma }}^{\alpha }=0\\ \left|\tau^{*\;\alpha }\right|=\tau_{c}^{\alpha }\Rightarrow {\dot{\gamma }}^{\alpha }\geq 0 \end{array}\right.$$

where $\tau^{*\;\alpha }$ is equal to $R^{*\;\alpha }:\sigma$, and $R^{*\;\alpha }$ is defined as follows:

(19) $$\forall \alpha =1,\ldots ,N_{s}:\;R^{*\;\alpha }=R^{\alpha }+\;R^{ns\alpha }$$

with $R^{ns\alpha }$ being the symmetric part of the non-Schmid orientation tensor $M^{ns\alpha }$, which can be easily deduced from Equation (17):

(20) $$\forall \alpha =1,\ldots ,N_{s}:\;M^{ns\alpha }=a_{1}({\vec{m}}^{\alpha }⊗{\vec{n}}_{1}^{\alpha })+a_{2}[({\vec{n}}^{\alpha }\times {\vec{m}}^{\alpha })⊗{\vec{n}}^{\alpha }]+a_{3}[({\vec{n}}_{1}^{\alpha }\times {\vec{m}}^{\alpha })⊗{\vec{n}}_{1}^{\alpha }]\;$$

The rate of the critical shear stresses is expressed by the following generic form:

(21) $$\forall \alpha =1,\ldots ,N_{s}:\;{\dot{\tau }}_{c}^{\alpha }=\sum_{\beta =1}^{N_{s}}h^{\alpha \beta }{\dot{\gamma }}^{\beta }\;$$

where h is a symmetric hardening matrix.

The time derivative of $\tau^{*\;\alpha }$ can be obtained after some straightforward calculations:

(22) $$\forall \alpha =1,\ldots ,N_{s}:\;{\dot{\tau }}^{*\;\alpha }=R^{*\;\alpha }:\sigma^{\nabla }$$

The expression of the co-rotational stress rate $\sigma^{\nabla }$ can be obtained by combining Equations (13) and (15)_(1)_:

(23) $$\sigma^{\nabla }=[C^{e}:d-\sigma \;Tr(d)]-\sum_{\alpha =1}^{N_{s}}{\dot{\gamma }}^{\alpha }\mathrm{sgn}(\tau^{\alpha })\;C^{e}:\;R^{\alpha }$$

Consequently, ${\dot{\tau }}^{*\;\alpha }$ can be expressed as follows:

(24) $$\forall \alpha =1,\ldots ,N_{s}:\;{\dot{\tau }}^{*\;\alpha }=R^{*\;\alpha }:[C^{e}:d-\sigma \;Tr(d)]-\sum_{\beta =1}^{N_{s}}{\dot{\gamma }}^{\beta }\;\mathrm{sgn}(\tau^{\beta })\;R^{*\;\alpha }:C^{e}:\;R^{\beta }$$

Let us now introduce the set A of active slip systems, which are defined as:

(25) $$\forall \alpha \in A:\;{\dot{\gamma }}^{\alpha }>0\;;\;{\dot{\tau }}^{*\;\alpha }\;\mathrm{sgn}(\tau^{*\;\alpha })-{\dot{\tau }}_{c}^{\alpha }=0$$

By using Equations (21) and (24), Equation (25) can be transformed as follows:

(26) $$\forall \alpha \in A:\;\sum_{\beta \in A}(\mathrm{sgn}(\tau^{*\;\alpha })\;\mathrm{sgn}(\tau^{\beta })\;R^{*\;\alpha }:C^{e}\;:R^{\beta }+h^{\alpha \beta })\;{\dot{\gamma }}^{\beta }=\mathrm{sgn}(\tau^{*\;\alpha })\;R^{*\;\alpha }:[C^{e}:d-\sigma \;Tr(d)]$$

The absolute values for the slip rates of the active slip systems can then be obtained from Equation (26):

(27) $$\forall \alpha \in A:\;{\dot{\gamma }}^{\alpha }=\sum_{\beta \in A}M^{\alpha \beta }\;\mathrm{sgn}(\tau^{*\;\beta })R^{*\;\beta }:[C^{e}:d-\sigma \;Tr(d)]$$

where M is the inverse of matrix P defined by the following index form:

(28) $$\forall \alpha ,\beta \in A:\;P^{\alpha \beta }=\mathrm{sgn}(\tau^{*\;\alpha })\;\mathrm{sgn}(\tau^{\beta })\;R^{*\;\alpha }:C^{e}:R^{\beta }+h^{\alpha \beta }$$

The nominal stress tensor n and the Cauchy stress tensor σ are related by the following relation:

(29) $$n=j\;f^{-1}\sigma$$

where j is the Jacobian of the microscopic deformation gradient f. The rate $\dot{n}$ of the microscopic nominal stress is determined by computing the time derivative of Equation (29):

(30) $$\dot{n}=j\;f^{-1}(\dot{\sigma }+\sigma \;Tr(d)-g.\sigma )$$

As an updated Lagrangian approach is used in the current investigation, Equation (30) can be reduced to the following form:

(31) $$\dot{n}=\dot{\sigma }+\sigma \;Tr(d)-g.\sigma$$

The latter can be related to ${\dot{\gamma }}^{\alpha }$ by combining Equations (9), (14) and (15):

(32) $$\dot{n}=C^{e}:d-\sigma .w-d.\sigma -\sum_{\alpha \in A}\left(C^{e}:R^{\alpha }+S^{\alpha }.\sigma -\sigma .S^{\alpha })\;{\dot{\gamma }}^{\alpha }sgn(\tau^{\;\alpha }\right).$$

The microscopic tangent modulus l can be obtained from Equations (27) and (32) after some lengthy but straightforward algebraic manipulations [16]:

(33) l=Ce−lσ1−lσ2−∑α∈A(sgn(τ α) (Ce:Rα+Sα.σ−σ.Sα)⊗∑β∈AMαβ sgn(τ* β)R* β:(Ce−σ⊗Id))

where Id stands for the second-order identity tensor, while lσ1 and lσ2 are fourth-order tensors, which are defined by convective terms of Cauchy stress components:

(34) lijklσ1=12 (δljσik−δkjσil) ; lijklσ2=12 (δikσlj+δilσkj)

By analyzing Equations (18) and (25), one can easily deduce that ${\dot{\gamma }}^{\alpha }$ is not determined by $\tau^{\alpha }$ and ${\dot{\tau }}^{\alpha }$ alone. In this case, the plastic flow is non-associated with the yield criterion. Indeed, stresses other than the Schmid stress (which is parallel to the slip) on that slip system enter the flow rule. Thereby, the normality rule is not respected. This phenomenon can be easily checked by analyzing Equation (15)_(1)_ and the definition of $\tau^{*\;\alpha }$. Indeed, we have:

(35) $$d^{p}\neq \sum_{\alpha =1}^{N_{s}}{\dot{\gamma }}^{\alpha }\;\mathrm{sgn}(\tau^{*\;\alpha })\;\frac{\partial \;\tau^{*\;\alpha }\;}{\partial \;\sigma }=\sum_{\alpha =1}^{N_{s}}{\dot{\gamma }}^{\alpha }\;\mathrm{sgn}(\tau^{*\;\alpha })\;R^{*\;\alpha }$$

Equation (35) implies that the plastic strain rate deviates from the direction of the outward normal to the yield surface (represented in stress space).

The effect of the non-associativity of the plastic flow on the decrease of the ductility limit has been largely studied in the literature with phenomenological models [11,31]. The aim of the current contribution is to numerically explore the effect of this non-associativity, considered at the single crystal scale, on the ductility limit of polycrystalline aggregates.

## 3. Algorithmic Aspects

The algorithm for the prediction of forming limit diagrams by the bifurcation approach is based on the two following nested loops:

- For each strain-path ratio ρ comprised between −1/2 and 1 (with typical intervals of 0.1).
  ■ For each time increment $I^{\Delta }=\left[t_{0},t_{0}+\Delta t\right]$:
    ✓ Compute the plane-stress tangent modulus $L^{\mathrm{PS}}$ from the 3D tangent modulus L by using an iterative procedure similar to the one developed in [16]. On the other hand, L is determined from the microscopic tangent moduli l of the different single crystals by Equation (7). Some indications on the method used to compute l are given after this algorithm.
    ✓ For θ=0° to 90°, at user-defined intervals (with typical increments of 1°):
      - compute the determinant of the acoustic tensor $\det(\vec{𝓝}.L^{\mathrm{PS}}.\vec{𝓝})=0,$. We recall that the components of $\vec{𝓝}$ are equal to (cosθ,sinθ).
    ✓ Search for the orientation that minimizes $\det(\vec{𝓝}.L^{\mathrm{PS}}.\vec{𝓝})=0,$ over the different values of θ. If $\det({\vec{𝓝}}_{\min}.L^{\mathrm{PS}}.{\vec{𝓝}}_{\min})\leq 0,$, then localized necking is reached. The corresponding angle θ is the orientation of the localization band, while the corresponding limit strain $E_{11}$ is equal to $\int_{0}^{t_{0}+\Delta t}G_{11}dt=t_{0}+\Delta t$ (as $G_{11}$ is equal to 1). The computation is then stopped. Otherwise, the integration is continued for the next time increment.

The microscopic tangent modulus l should be computed at $t_{0}+\Delta t$ by using Equation (33). As clearly demonstrated in Equations (33) and (34), the different microscopic variables should be updated to compute l. The update of these variables is based on an implicit integration scheme over $I^{\Delta }$. This integration scheme belongs to the family of ultimate algorithms and is similar to the one developed in [32] for the classical Schmid law. The novelty in the algorithm used here, compared to the algorithm of [32], consists in the addition of the non-Schmid effects. By carefully analyzing the constitutive equations at the single crystal scale, one can deduce easily that the determination of the set of active slip systems, from the set of potentially active slip systems $P\;(=\{\alpha =1,\ldots ,N_{s}:\left|\tau^{*\;\alpha }(t_{0})\right|=\tau_{c}^{\alpha }(t_{0})\}$), as well as the corresponding slip rates ${\dot{\gamma }}^{\alpha }$ allows the determination of all other mechanical variables. In this objective, a combinatorial search strategy, analogous to the one proposed in [32], is adopted to determine the set of active slip systems from the set of potentially active slip systems. This search strategy is performed iteratively and, at each iteration, a subset of the set of potentially active slip systems is chosen to be the set of active slip systems. The slip rates corresponding to the assumed set of active slip systems are calculated by using Equation (27). If matrix P (Equation (28)) is singular (which corresponds to the widely-known indetermination problem), the pseudo-inversion technique is adopted to invert it and then to compute the slip rates of the active slip systems. For the other slip systems, their slip rates are assumed to be equal to zero. After this step, the generalized Schmid law defined by Equation (18) is assessed for all of the potentially active slip systems. If at least one constraint of this generalized Schmid law is not satisfied, then the assumed set is not an effective set of active slip systems and another set is chosen. It must be noted that, due to the introduction of the non-Schmid effects, matrix P is not symmetric. The asymmetry of matrix P leads to a slight increase in the computational effort spent by the solver. Once the slip rates of the active slip systems are computed, the other mechanical variables (the Cauchy stress tensor σ, the rotation of the lattice frame r) should be updated, and the microscopic tangent modulus l is computed.

## 4. Numerical Results

### 4.1. Material Data

**Figure 1:**
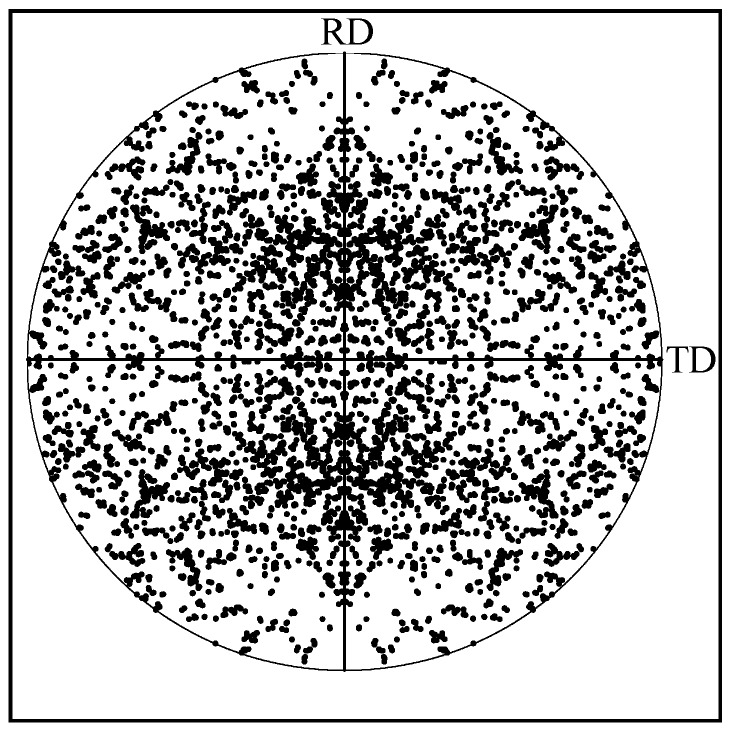
Initial crystallographic texture: {111} pole figure.

In this section, we examine the impact of the non-Schmid effects on the constitutive response at the single crystal scale (Section 4.2) and on the prediction of the onset of strain localization at the polycrystal scale (Section 4.3). A polycrystalline aggregate made of 1000 single crystals is considered. The initial crystallographic texture corresponding to this polycrystalline aggregate is randomly generated, as shown in Figure 1.

The initial state for each single crystal, in terms of stress and crystallographic slip, is defined as:

(36) $$\sigma (t=0)=0\;;\;\forall \alpha =1,\ldots ,12:\;\gamma^{\alpha }(t=0)=0\;$$

The hardening law used in this work has been initially introduced in [33]. In this law, the hardening matrix h involved in Equation (21) is given by the following index expression:

(37) $$\forall \alpha ,\beta =1,\ldots ,24:\;h^{\alpha \beta }=\hat{h}(A)\;[q+(1-q)\;\delta^{\alpha \beta }]\;;\;\hat{h}(A)=h_{0}\;{\left(1+\frac{h_{0}\;A}{n\;\tau_{0}}\right)}^{n-1}$$

where $\delta^{\alpha \beta }$ is the Kronecker symbol and A is the sum of the accumulated plastic slip on all slip systems. However, n, $\tau_{0}$, q and $h_{0}$ are material parameters. The values of the parameters relating to elasticity and hardening are reported in Table 1.

To extensively analyze the influence of the non-Schmid effects on the material response and formability limit prediction, it is desirable to set up a sensitivity study where the non-Schmid parameters $a_{1}$, $a_{2}$, and $a_{3}$ (see Equation (17)) would be varied simultaneously or separately in the range of their admissible values. Here, and for the sake of brevity, attention is restricted to the effect of parameter $a_{1}$ on the numerical predictions.

### 4.2. Significance of the Non-Schmid Effects on the Microscale Constitutive Response

To analyze the significance of the non-Schmid effects on the microscopic mechanical behavior, let us apply a plane-stress loading to a single crystal. This loading is defined by the following microscopic velocity gradient:

(38) $$g=\left(\begin{array}{ccc} 1 & 0 & ?\\ 0 & -0.5 & ?\\ ? & ? & ? \end{array}\right)$$

where the unknown components of g ($g_{13},g_{23},g_{31},g_{32}$, and $g_{33}$) are deduced by the following plane-stress condition:

(39) $${\dot{n}}_{13}={\dot{n}}_{23}={\dot{n}}_{31}={\dot{n}}_{32}={\dot{n}}_{33}=0\;$$

The initial orientation matrix of the studied single crystal is assumed to be equal to the identity tensor.

**Figure 2:**
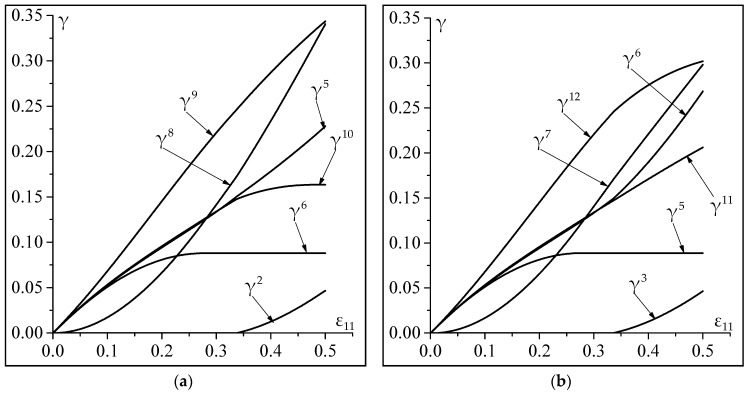
Significance of the non-Schmid effects on the slip system activity: (**a**) Results without non-Schmid effects ($a_{1}=a_{2}=a_{3}=0$); (**b**) Results with non-Schmid effects ($a_{1}=0.2;a_{2}=a_{3}=0$).

The impact of the non-Schmid effects on the evolution of the accumulated plastic slip of the activate slip systems $\gamma^{\alpha }(=\int_{0}^{t}\left|{\dot{\gamma }}^{\alpha }\right|\;dt)$, as a function of $\varepsilon_{11}(=\int_{0}^{t}g_{11}\;dt)$, is depicted in Figure 2. For the case of classical Schmid law, the parameters $a_{1}$, $a_{2}$, and $a_{3}$ are obviously set to 0. This figure shows that the activity of the slip systems and the evolution of the corresponding accumulated plastic slips are strongly influenced by the value of parameters $a_{1}$, $a_{2}$, and $a_{3}$. Indeed, when the non-Schmid effects are not considered, systems 2, 5, 6, 8, 9, 10 are activated during loading. By contrast, systems 3, 5, 6, 7, 11, 12 are activated when the non-Schmid effects are considered in the constitutive modeling. This result is expected, considering the influence of parameters $a_{1}$, $a_{2}$ and $a_{3}$ on the values of the components of matrix P (see Equation (28)). This matrix clearly affects the activity of the slip systems and the evolution of the corresponding accumulated plastic slips (see Equation (27)).

**Figure 3:**
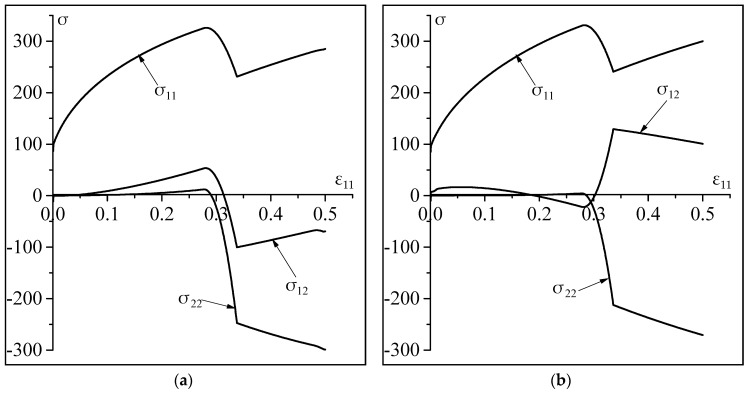
Impact of the non-Schmid effects on the evolution of the in-plane components of the Cauchy stress tensor: (**a**) Results without non-Schmid effects ($a_{1}=a_{2}=a_{3}=0$); (**b**) Results with non-Schmid effects ($a_{1}=0.2;a_{2}=a_{3}=0$).

The above-observed difference in the activity of the slip systems induces a difference in the evolution of the in-plane components of the Cauchy stress tensor, as shown in Figure 3. The abrupt changes observed on the evolution of the components of the stress tensor are explained by the change in the activity of the slip systems.

### 4.3. Influence of the Non-Schmid Effects on Localized Necking

**Figure 4:**
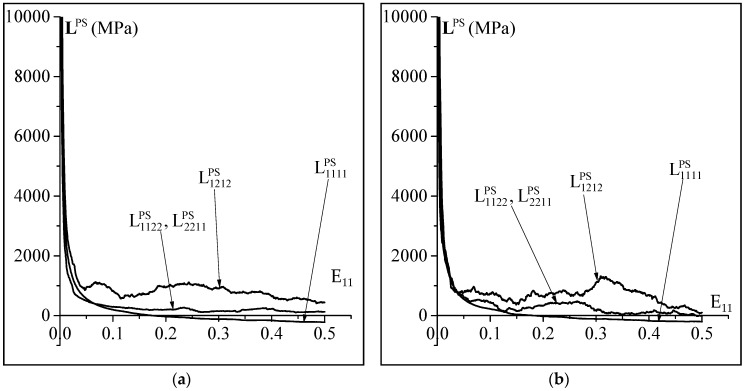
Significance of the non-Schmid effects on the evolution of some representative components of the macroscopic tangent modulus for the plane-strain state: (**a**) Results without non-Schmid effects ($a_{1}=a_{2}=a_{3}=0$); (**b**) Results with non-Schmid effects ($a_{1}=0.2;a_{2}=a_{3}=0$).

As previously mentioned, the occurrence of plastic strain localization is predicted by the bifurcation theory. This theory states that bifurcation takes place when the determinant of the acoustic tensor becomes equal to zero (Equation (4)). Hence, to analyze the impact of the non-Schmid effects on plastic strain localization, let us first analyze their impact on the evolution of some components of the 2D macroscopic tangent modulus $L^{\mathrm{PS}}$. To this aim, the evolution of the components $L_{1111}^{\mathrm{PS}}$, $L_{1122}^{\mathrm{PS}}$, $L_{2211}^{\mathrm{PS}}$ and $L_{1212}^{\mathrm{PS}}$, as function of $E_{11}(=\int_{0}^{t}G_{11}\;dt)$ for the plane-strain state, is plotted in Figure 4. This figure clearly shows that the value of the non-Schmid coefficient $a_{1}$ has a significant effect on the evolution of the components of $L^{\mathrm{PS}}$. Furthermore, the consideration of the non-Schmid effects leads to an important fluctuation of the evolution of the components of $L^{\mathrm{PS}}$. This observation is attributed to the fact that the normality of the plastic flow to the yield function is not respected when the non-Schmid effects are accounted for. As clearly shown in Figure 4, all of the components of $L^{\mathrm{PS}}$ decrease during loading including also, in particular, the shearing component $L_{1212}^{\mathrm{PS}}$. This feature is an obvious consequence of the multi-slip nature of crystal plasticity, which results in the formation of vertices at the current points of the single crystal yield surfaces. Note that the decrease of the shearing components of the tangent modulus is the most important destabilizing factor responsible for bifurcation, thus leading to early plastic strain localization. It is also important to notice that the difference between $L_{1122}^{\mathrm{PS}}$ and $L_{2211}^{\mathrm{PS}}$ does not exceed 1%, which explains why the curves representing these tangent modulus components are indistinguishable, as clearly observed in Figure 4.

**Figure 5:**
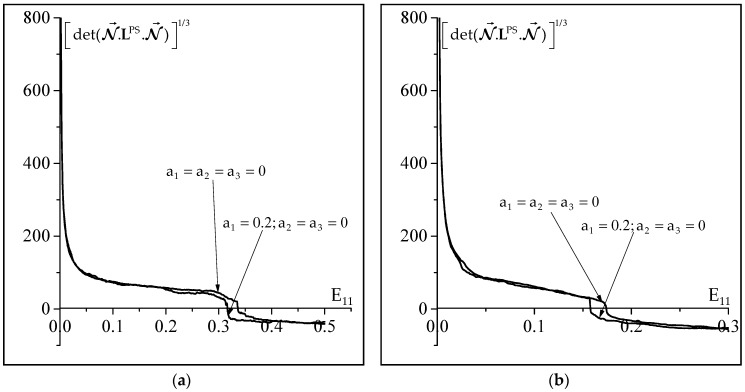
Impact of the non-Schmid effects on the evolution of the minimum of the determinant of the acoustic tensor as a function of E_11_: (**a**) Uniaxial tensile state (ρ=−0.5); (**b**) Plane-strain state (ρ=0).

The influence of the non-Schmid effects on the initiation of plastic strain localization for two strain paths (uniaxial tension and plane-strain tension) is investigated in Figure 5, where the minimum of the determinant of the acoustic tensor is plotted as a function of E_11_. The results of this figure clearly show that the non-Schmid effects tend to precipitate the occurrence of localized necking. Indeed, the limit strains predicted when the non-Schmid effects are considered are lower than those determined by the classical Schmid law, by about 3% of deformation.

**Figure 6:**
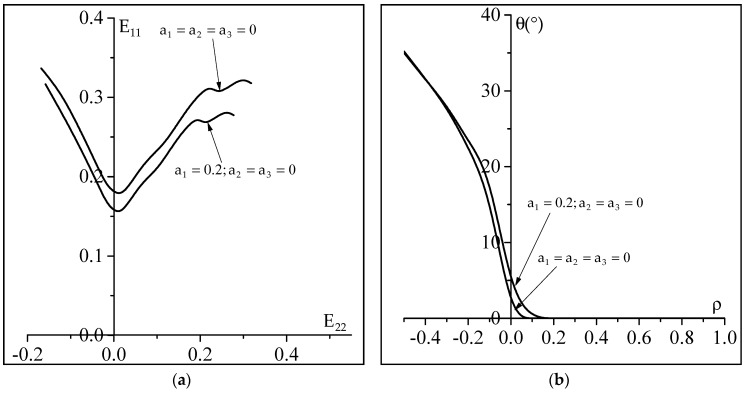
Impact of the non-Schmid effects on: (**a**) the shape and the level of the forming limit diagrams; (**b**) the evolution of the necking band orientation as a function of the strain-path ratio.

The results obtained in Figure 5 are generalized to the whole range of strain paths in Figure 6a. This latter figure shows that the forming limit diagram predicted by the classical Schmid law is higher than its counterpart, which is predicted when the non-Schmid effects are accounted for. The impact of the non-Schmid effects on the necking band orientation is analyzed in Figure 6b. This latter figure shows that the non-Schmid effects have a slight influence on the necking band orientation. Also, the band inclination angle is found to be equal to 0 for strain-path ratios larger than 0.1, for both plastic flow rules (i.e., the classical Schmid law and the one which accounts for non-Schmid effects).

**Figure 7:**
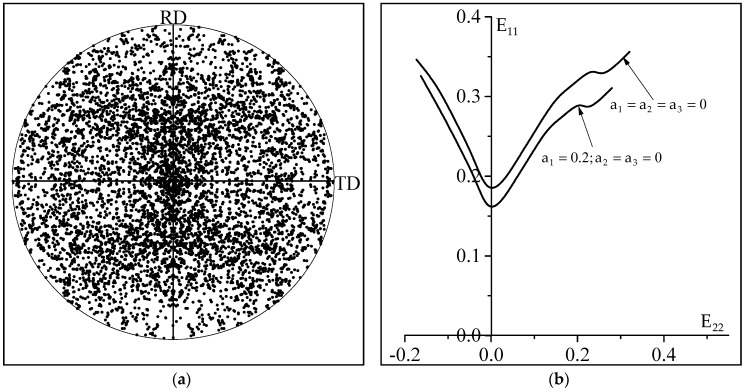
(**a**) Initial crystallographic texture ({111} pole figure); (**b**) Predicted forming limit diagrams.

The effect of the crystallographic texture on the ductility of polycrystalline sheet metals has been analyzed in several contributions (see for instance, references [16,34]). It has been demonstrated in earlier contributions that the predicted limit strains remain almost insensitive to the initial texture in the range of negative strain paths. By contrast, in the range of positive strain paths, the opposite trend is observed. In fact, both the shape and the overall level of the predicted FLDs are highly sensitive to the initial crystallographic texture. To further investigate the influence of the non-Schmid effects on the ductility limit, we enlarge the analysis by adopting a new crystallographic texture (see Figure 7a), in order to compare the corresponding FLDs, predicted with and without considering the non-Schmid effects. The FLDs predicted using this new texture are reported in Figure 7b. The material parameters used to obtain the predictions of Figure 7b are the same as those used for Figure 6a (except for the initial crystallographic texture). The conclusion of these new comparisons, associated with the new crystallographic texture, is the same as that revealed by Figure 6: the non-Schmid effects tend to reduce the ductility limit for the whole range of strain paths. A more detailed study will be conducted in future works to further investigate and to analyze the combined influence of the crystallographic texture and the non-Schmid effects.

## 5. Concluding Remarks

In the current contribution, we have analyzed the impact of the consideration of the non-Schmid effects on the mechanical behavior and on the onset of plastic strain localization in BCC polycrystalline materials. In the proposed model, the non-Schmid effects are accounted for at the single crystal scale by introducing additional terms in the expression of the resolved shear stress. Three scalar parameters ($a_{1}$, $a_{2}$ and $a_{3}$) have been used to account for these non-Schmid effects. The ductility limit is predicted by coupling the full-constraint Taylor scale-transition scheme to the bifurcation theory. The influence of the non-Schmid effects, through the variation of parameter $a_{1}$ on the prediction of plastic strain localization is particularly analyzed. The numerical simulations reveal that the consideration of non-Schmid effects ($a_{1}\neq 0$) tends to precipitate the onset of localized necking. The present investigation will be extended in future works to other multiscale schemes, which are more relevant than the Taylor multiscale scheme, such as the self-consistent approach and the periodic homogenization technique. An extensive study will be carried out in future works, which will adopt more elaborate non-Schmid models, to better analyze the impact of the non-Schmid effects on the prediction of plastic strain localization.

## Figures and Tables

**Table 1:** Material parameters.

Elasticity		Hardening			
E	ν	n	$\tau_{0}$	q	$h_{0}$
65 GPa	0.3	0.15	40 MPa	1.4	390 MPa

## Appendix A

### Slip Systems for the BCC Single Crystals Used in the Non-Schmid Crystal Plasticity Model

For BCC single crystals, the number of crystallographic slip systems is equal to 24:12 slip systems of the family {110}〈111〉 and 12 slip systems of the family {112}〈111〉. However, attention is focused here on the {110}〈111〉 slip systems with non-Schmid effects, since the geometry of 1/2〈111〉 screw dislocations on the {110} planes has been better understood in experiments and atomistic simulations at low temperatures, which is of particular interest in this work. Each slip system is characterized by three orthonormal vectors $\left({\vec{m}}^{\alpha },{\vec{n}}^{\alpha },{\vec{n}}_{1}^{\alpha }\right)$ in the deformed configuration. ${\vec{m}}^{\alpha }$ is the vector parallel to the slip line, ${\vec{n}}^{\alpha }$ is the vector normal to the slip plane and ${\vec{n}}_{1}^{\alpha }$ is the normal to the plane $\left\{1\bar{1}0\right\}$ in the slip direction zone, which also forms an angle of −60° with ${\vec{n}}^{\alpha }$. In the intermediate configuration, these vectors are defined by the following relations:

(A1) $$\forall \;\alpha =1,\ldots ,24:\;\left\|{\vec{m}}_{{}^{0}}^{\alpha }\right\|=\left\|{\vec{n}}_{{}^{0}}^{\alpha }\right\|=\left\|{\vec{n}}_{{}^{10}}^{\alpha }\right\|=1\;;\;{\vec{m}}_{{}^{0}}^{\alpha }.{\vec{n}}_{{}^{0}}^{\alpha }=0$$

Vectors ${\vec{m}}_{{}^{0}}^{\alpha }$, ${\vec{n}}_{{}^{0}}^{\alpha }$, and ${\vec{n}}_{{}^{10}}^{\alpha }$ are given in Table A1.

**Table A1:** List of slip systems for a BCC crystallographic structure [28].

α	**1**	**2**	**3**	**4**	**5**	**6**
$\sqrt{3}\;{\vec{m}}_{{}^{0}}^{\alpha }$	$\left[1\bar{1}1\right]$	$\left[\bar{1}\bar{1}1\right]$	[111]	$\left[\bar{1}11\right]$	$\left[\bar{1}11\right]$	$\left[\bar{1}\bar{1}1\right]$
$\sqrt{2}\;{\vec{n}}_{{}^{0}}^{\alpha }$	[011]	[011]	$\left[0\bar{1}1\right]$	$\left[0\bar{1}1\right]$	[101]	[101]
$\sqrt{2}\;{\vec{n}}_{{}^{10}}^{\alpha }$	$\left[\bar{1}10\right]$	$\left[0\bar{1}1\right]$	$\left[10\bar{1}\right]$	$\left[\bar{1}\bar{1}0\right]$	[101]	$\left[01\bar{1}\right]$
α	**7**	**8**	**9**	**10**	**11**	**12**
$\sqrt{3}\;{\vec{m}}_{{}^{0}}^{\alpha }$	[111]	$\left[1\bar{1}1\right]$	$\left[\bar{1}11\right]$	$\left[\bar{1}1\bar{1}\right]$	[111]	$\left[11\bar{1}\right]$
$\sqrt{2}\;{\vec{n}}_{{}^{0}}^{\alpha }$	$\left[\bar{1}01\right]$	$\left[\bar{1}01\right]$	[110]	[110]	$\left[\bar{1}10\right]$	$\left[\bar{1}10\right]$
$\sqrt{2}\;{\vec{n}}_{{}^{10}}^{\alpha }$	$\left[1\bar{1}0\right]$	[011]	$\left[\bar{1}0\bar{1}\right]$	[110]	$\left[\bar{1}01\right]$	$\left[0\bar{1}\bar{1}\right]$
